# The adult ADHD assessment quality assurance standard

**DOI:** 10.3389/fpsyt.2024.1380410

**Published:** 2024-08-02

**Authors:** Marios Adamou, Muhammad Arif, Philip Asherson, Sally Cubbin, Laurence Leaver, Jane Sedgwick-Müller, Ulrich Müller-Sedgwick, Kobus van Rensburg, James Kustow

**Affiliations:** ^1^ School of Human and Health Sciences, University of Huddersfield, Huddersfield, United Kingdom; ^2^ Adult ADHD Clinic, South West Yorkshire Partnership National Health Service (NHS) Foundation Trust, Huddersfield, United Kingdom; ^3^ Adult ADHD Clinic, Leicestershire Partnership National Health Service (NHS) Trust, Leicester, United Kingdom; ^4^ Social Genetic and Developmental Psychiatry, King’s College London, London, United Kingdom; ^5^ Adult ADHD Clinic, Manor Hospital, Oxford, United Kingdom; ^6^ Green Templeton College, Oxford, United Kingdom; ^7^ Health and Community Services, Government of Jersey, St Helier, Jersey; ^8^ Faculty of Nursing, Midwifery and Palliative Care, King’s College London, London, United Kingdom; ^9^ Department of Psychiatry, University of Cambridge, Cambridge, United Kingdom; ^10^ Adult ADHD Service, Barnet, Enfield and Haringey Mental Health National Health Service (NHS) Trust, London, United Kingdom; ^11^ Private Practitioner, Northampton, United Kingdom

**Keywords:** adult attention deficit hyperactivity disorder, adult ADHD, practice guidelines, diagnostic assessment, quality standard assessment

## Abstract

**Background:**

Attention Deficit Hyperactivity Disorder (ADHD) frequently persists into adulthood. There are practice guidelines that outline the requirements for the assessment and treatment of adults. Nevertheless, guidelines specifying what constitutes a good quality diagnostic assessment and report and the competencies required to be a specialist assessor are lacking. This can lead to variation in the quality and reliability of adult ADHD assessments. Poor quality assessments may not be accepted as valid indicators of the presence of ADHD by other clinicians or services, resulting in wasteful re-assessments and delays in providing treatment. To address this issue the UK Adult ADHD Network (UKAAN) proposes a quality framework for adult ADHD assessments - the Adult ADHD Assessment Quality Assurance Standard (AQAS).

**Methods:**

The co-authors agreed on five questions or themes that then guided the development of a set of consensus statements. An initial draft was reviewed and amended in an iterative process to reach a final consensus.

**Results:**

What constitutes a high-quality diagnostic assessment and report was agreed by consensus of the co-authors. The resulting guideline emphasises the need to evaluate impairment, describes core competencies required by the assessor and highlights the importance of linking the diagnosis to an appropriate post-diagnostic discussion. Assessments should be completed in the context of a full psychiatric and neurodevelopmental review, and need good interview skills, using a semi-structured interview with open questioning and probing to elicit real life examples of symptoms and impairments. It is recommended that 2 hours or more is required for an adequate assessment including both the diagnostic assessment and initial post-assessment discussions.

**Conclusion:**

The AQAS has been developed as a practical resource to support reliable and valid diagnostic assessments of adult ADHD. It is intended to complement formal training. A secondary objective is to empower patients by providing them with evidence-based information on what to expect from an assessment and assessment report.

## 1 Introduction

Epidemiological data indicates that 3-4% of the general adult population meet diagnostic criteria for attention deficit hyperactivity disorder (ADHD) (1). For many years, ADHD was thought to be a condition restricted to childhood, and this idea was pervasive until the 1990s (2). The existence of ADHD in adults has historically been challenged, with symptoms being attributed to other conditions, disregarded altogether, or explained away as common behaviours that are also experienced by the neurotypical population (3). Thankfully, this is now a peripheral view that is strongly challenged by the extensive body of evidence relating to the condition (4–7).

The two main classification systems used for the diagnosis of mental health conditions are the International Classification of Diseases (ICD-11) and the Diagnostic and Statistical Manual of Mental Disorders (DSM-5-TR). The ICD-11 and DSM-5-TR are now broadly similar in their classification of ADHD in adults, and both are approved for use. DSM’s fourth edition (DSM-IV) was the first to highlight that adults may meet the criteria for a diagnosis of ADHD (8), with greater emphasis and further specification of the common features in adulthood being added in the fifth edition (DSM-5) (9).

A recent review called for a lifespan perspective of ADHD and highlighted considerable heterogeneity in reports of its persistence into adulthood; estimates ranging from 15-80% (5), depending on the population investigated and the method of assessment. The relatively recent widespread recognition that ADHD often persists across the lifespan, has left adult service providers working out how best to meet this need, that encompasses up to three generations of undiagnosed adults with ADHD. Various models of care have emerged and are still evolving (10, 11), but to date the relatively few established adult ADHD services within the National Health Service (NHS) in the United Kingdom (UK) have generally not been resourced sufficiently to meet the considerable need (12, 13). This problem likely exists in many other countries.

Evidence based guidelines for the diagnosis and management of adult ADHD have been developed by the National Institute of Health and Care Excellence (NICE) (14–16) the British Association for Psychopharmacology (BAP) (17), and The Royal College of Psychiatrists in Scotland (18), among others. These guidelines outline the requirements for a diagnostic assessment and treatment, but they do not specify detailed procedures guiding how this should be done in practice. Until recently many psychiatric trainees will not have been taught about adult ADHD, or how to assess or manage it.

Since its inception in 2009, the UK Adult ADHD Network (UKAAN) has sought to promote the highest standards of care for adults with ADHD, and influence practice both nationally and internationally. UKAAN has trained several thousands of clinicians, from the UK and worldwide in the assessment and treatment of ADHD in adults, and is highly respected for the quality of its training programmes.

Using a consensus approach among UKAAN executive committee members, for the first time, we have developed detailed practice guidelines for the assessment and reporting of adult ADHD; named the Adult ADHD Assessment Quality Assurance Standard (AQAS). The AQAS has been informed by rich discussion and debate between experienced service providers and trainers, many of whom lead well-established adult ADHD services in the UK. The AQAS primarily aims to define standards for the diagnostic assessment of adult ADHD. Its purpose is to improve the quality, consistency, and accuracy of the assessment, and it is designed to serve practitioners working across various settings (e.g., specialist ADHD clinics, as well as those in general secondary mental health services, private practice, and higher education), as well as service users who include patients and their family members and informal carers.

### 1.1 Why is the AQAS needed?

Despite considerable improvements in the diagnosis and management of ADHD in recent years, assessment and treatment protocols for adults with ADHD are not well established, and less regulated than those for children and adolescents (19). A recent study revealed significant geographical variations in the quality and provision of care offered to adults with ADHD in the UK, with only 27% of services providing the full range of services recommended by NICE (20). Furthermore, in many cases transition from child to adult services is poorly managed, and more generally many adults with ADHD are not receiving adequate or appropriate care (21).

In 2008 NICE published the first clinical guideline in the UK that included adults with ADHD (14). The guideline identified that the “assessment and treatment of ADHD is currently delivered by a range of practitioners with very variable levels of training and competence”. Indeed, in the UK the lack of appropriately trained and experienced practitioners and/or assessors has contributed to a bottleneck of adults waiting to access assessment and treatment (22). Neither the Competency Based Curriculum for Specialist Training in Psychiatry produced by the Royal College of Psychiatrists (23) nor the British Psychological Society standards for doctoral programs in clinical or educational psychology (24, 25), require competence in adult neurodevelopmental disorders including ADHD. Although evaluation of neurodevelopmental disorders was added as a core requirement in the 2022 curriculum for psychiatrists in the UK, specific points about ADHD assessment and treatment were not included (26).

An additional concern has been the impact of the COVID-19 pandemic, which may be linked to a worsening of the core symptoms of ADHD and increased burden on adults with ADHD and their families (27). Adults with ADHD and other common mental health disorders are particularly vulnerable to distractibility, restlessness and problems with organisation and planning. Therefore, lockdown requirements during the pandemic, and changes to greater working at home with less structure and daily supervision may have had an overall negative impact on their wellbeing. There were also concerning reports about behaviours related to ADHD leading to a greater risk of spreading COVID-19 infection, together with inadequate access to ADHD services for new assessments and follow-ups (28). Related to this, the authors of this report have observed a considerable surge in referrals for ADHD assessments during the COVID-19 pandemic, paralleling that seen in other areas of mental health (29). The higher rates of referral appear to have continued beyond the pandemic, greatly increasing the pressures on ADHD and mental health services more generally.

The reasons behind the increase in ADHD referrals is a matter for conjecture but most likely they are multifactorial. They may represent greater public awareness of ADHD (22, 30), an increasing tendency to attribute as well as misattribute symptoms of other mental illnesses to ADHD, and a rapid shift towards lone-working and home working practices without appropriate support. These factors are known to have had a destabilising effect on people’s mental health and coping mechanisms (31).

In our experience, an increasing problem of inaccurate diagnoses of adult ADHD has also emerged. The potential for misattributing symptoms of ADHD to other mental health disorders, and conversely misattributing other mental health symptoms to ADHD, applies both to members of the public, as well as practitioners who may not have adequate knowledge or expertise in the assessment of ADHD. In the past, it was largely a problem of the former, but now increasingly the latter is being observed by the co-authors. The potential for misattribution is understandable because core features of ADHD such as inattention, distractibility, restlessness and emotional dysregulation are a feature of other common mental health disorders (32).

A further challenge is that over 60% of those with adult ADHD present with one or more comorbid disorders (1). The comorbidities are wide ranging but commonly include anxiety and mood disorders, substance abuse disorders (1, 33), autism (34, 35), bipolar disorder (1), personality disorders (1, 33), post-traumatic stress disorder (36) and (37) Tourettes’s syndrome (38). A history of traumatic life events is not uncommon in ADHD and is associated with greater impairment and comorbidities (36, 39, 40). Specific reading difficulties (dyslexia) (41) and developmental coordination disorder (dyspraxia) (42), that are often identified in educational settings and are not typically dealt with by adult mental health services, are also commonly seen to co-occur in ADHD. There may also be somatic or physical health comorbidities including obesity (43), asthma (44), type II diabetes (45), fibromyalgia (46), joint hypermobility (47), chronic fatigue and migraine, that can further complicate the diagnostic picture.

To unravel these complexities and come to an accurate diagnosis of ADHD, diagnostic assessments need to consider the presence of symptoms across the lifespan, functional impairments, and a detailed evaluation of other mental health, neurodevelopmental and somatic conditions. This requires an appropriately trained and competent practitioner or assessor.

Misdiagnosis, in either direction, can delay the initiation of appropriate pharmacological and/or non-pharmacological interventions. Misdiagnosing ADHD for other conditions will result in individuals with ADHD not receiving effective targeted treatments. Undiagnosed, untreated or poorly managed ADHD, can lead to inappropriate treatment with other psychotropic medications (commonly antidepressants, antipsychotics and mood stabilisers), and persistent functional and psychosocial impairments (48), including academic, employment and relationship difficulties (49). Then there is the negative impact to mental and physical well-being if ADHD is wrongly diagnosed and treated.

With the proliferation of easily available online and remote assessments (50) the provision of adult ADHD assessments has rapidly increased, particularly in the private sector. This has led to mental health services with considerable heterogeneity in their approach and the quality of care. Allied to this are broader concerns about the inappropriate medicalisation of the milder (non-clinical) end of the neurodiversity spectrum.

Due to the wide variation in service provision and assessor competency, general practitioners and mental health professionals may encounter adults with ADHD, who have had very different assessment and management experiences, with considerable variability in the quality of ADHD assessment reports. The ‘good practice in prescribing and managing medicines and devices’ produced by the General Medical Council (51) advises that clinicians ‘must only prescribe if it is safe to do so’ and adds, that it is ‘not safe to prescribe if you do not have sufficient information about the patient’s health, or if the mode of consultation is unsuitable to meet their needs’.

If the quality of information captured in an ADHD assessment report is deemed inadequate, it is common practice to subject the individual to further assessments and unacceptably long waiting lists. Apart from the inefficient use of resources and unacceptable treatment delays, this generates distress in the patient, especially when the diagnostic outcomes of the two assessments differ.

By establishing agreed quality standards for adult ADHD diagnostic assessments and assessment reports, variances in quality can be narrowed and overall care improved. We intend for the AQAS to serve as a benchmark that ensures patients receive reliable and valid diagnostic assessments, assessment reports and treatment plans for adult ADHD, reflecting high quality in clinical practice and standards of care. The AQAS recommendations should also facilitate more streamlined continuity of care and shared care arrangements for adult ADHD, particularly when transitioning from child to adult services, and between private and NHS services.

## 2 Methods

### 2.1 The UK Adult ADHD Network (UKAAN)

Co-authors of this guideline are the current UKAAN executive committee members. UKAAN was founded in 2009, following publication of the NICE ADHD guidelines in 2008, by a group of experienced mental health specialists who were delivering clinical services for adults with ADHD within the UK National Health Service (NHS). The overarching aim of UKAAN has been to support the development of clinical services for adult ADHD, and provide training for practioners working in adult mental health, neurodisability and educational settings. Among the co-authors there is a combined experience of working with over 20,000 adults with ADHD, over 130 clinician years. Today, some members of UKAAN’s executive and training committees, and regional leads, are also active in private practice, run adult ADHD services in other parts of the British Isles and have academic affiliations with UK universities. With its finger on the pulse in terms of optimal clinical practice, the organisation is in an ideal position to generate a set of quality standards.

### 2.2 Development of the AQAS

In response to the concerns outlined above, the executive committee of UKAAN met in May 2021 to discuss the development of guidelines for good clinical practice for adult ADHD diagnostic assessments and assessment reports. Initial discussions focused on delineating the problem. There was a clear consensus regarding the considerable variation in quality of adult ADHD diagnostic assessments and assessment reports across the UK, and the need to establish quality standards that practitioners can work.

AQAS was developed through vigorous discussion of a series of agreed assessment-related questions and themes. These discussions used the UK NICE guideline recommendations as the basis for good clinical practice in diagnosis and treatment of adult ADHD (14–16) that AQAS is designed to support. An iterative process was followed through drafting and redrafting, culminating in the formulation of a set of quality standards, or consensus statements, supported by all co-authors. This approach concords well with the phenomenological method used to gain an ‘insiders’ or emic perspective of clinical experiences, knowledge, and expertise (52).

An initial draft of AQAS was circulated to all the UKAAN regional leads, as well as the executive committee, for their review and comments, with the aim of achieving a broad consensus. The regional leads submitted detailed responses to the first draft, that was then amended, and a second draft produced. Following a series of rigorous reviews by the co-authors, with over ten further iterations, a final draft of AQAS was produced.

In developing this quality standard, the AQAS identifies three levels of recommendations: essential, highly recommended, and optional. Those that are considered essential are non-negotiable. These include existing recommendations from the NICE guidelines and the application of DSM criteria. Those that are ‘highly recommended’, represent what the authors believe to be a minimum standard of quality care that should be followed in most assessments. Those that are optional are highly desirable but not considered essential for practice.

As adult ADHD impairments range from relatively mild (including both high and low functioning individuals) to severely disabling and complex (often including comorbidity with other neurodevelopmental, mental health, and somatic conditions or disorders), the authors felt it was important to differentiate between the assessment of a relatively ‘straightforward’ case of adult ADHD (more common in educational settings), and the assessment of a ‘more complex’ case (more common in specialist adult ADHD clinics). The quality standards described in the AQAS encompass the assessment of both ‘straightforward’ and ‘more complex’ cases of adult ADHD, but there will clearly be nuances in approach.

### 2.3 Terminology used in the AQAS

The AQAS refers to individuals presenting for an adult ADHD assessment in clinical settings as ‘patients’. The use of terms such as ‘service user’ and ‘client’ are considered more appropriate in non-medical settings such as educational institutions or psychotherapy practices where individuals with ADHD may seek support. We also note the more inclusive nature of the term service user that helpfully encompasses patients, family members and informal carers. Here we consider the term patient to be best suited for the AQAS with its primary focus being on diagnostic assessments in clinical settings.

AQAS uses the word ‘screening’ when considering the presence or absence of another condition. Screening can range from a brief enquiry to the use of more formal screening tools. We are leaving it to clinical judgement as to the level of screening employed.

### 2.4 The key questions and themes addressed by the AQAS

Five questions and themes were debated, as follows:

1. What constitutes a ‘gold-standard’ adult ADHD diagnostic assessment?
2. What information should be included in an adult ADHD assessment report?
3. What levels of impairment are required to make a diagnosis of adult ADHD?
4. How long should an adult ADHD diagnostic assessment take?
5. What core competencies are required to conduct a diagnostic assessment of adult ADHD?

## 3 The Adult ADHD Assessment Quality Assurance Standard (AQAS, 2023)

### 3.1 Theme 1: What constitutes a ‘gold-standard’ adult ADHD diagnostic assessment?

Currently there are no biomarkers, cognitive or neuroimaging tests with sufficient specificity and sensitivity to diagnose ADHD. The gold standard for an adult ADHD diagnostic assessment is a detailed exploration of the clinical and behavioural presentation, using a semi-structured diagnostic interview, supported by supplementary and/or collateral information (53). This approach is endorsed by international consensus and guidelines that recommend the use of reliable and validated diagnostic clinical interviews (54).

The semi-structured interview should carefully evaluate the presence or absence of each of the 18 DSM-5-TR (or ICD-11) symptoms, both currently (in adulthood) and retrospectively (in childhood). Additional criteria required for the diagnosis include the presence of functional impairments, onset of several symptoms in childhood, and pervasiveness across different settings. It is important that this interview is not conducted as a simple tick box exercise, with binary “yes” or “no” answers, regarding the presence or absence of each symptom. The requirement is for a detailed explorative interview.

The assessor should make use of open-ended questions to elicit information, along with careful probing, with real-life examples. The individual should be encouraged to speak freely about their concerns and difficulties associated with ADHD, and through this the assessor is able to form their own clinical impression and judge whether a core symptom of ADHD is present or absent.

ADHD symptoms need to be persistent, pervasive and lead to problems in daily life. Reflecting the neurodevelopmental nature of ADHD, symptoms are ‘trait-like’ (persisting over time, with at least several symptoms being present since childhood). They should not be episodic, reflecting change from a pre-morbid baseline, that is usually the hallmark of later-onset episodic disorders. They may however fluctuate throughout the day, depending on the engagement of individuals with different tasks and situations. For example, distractibility and restlessness may be reduced when engaged in highly salient activities. To meet diagnostic criteria, symptoms must also be pervasive (occurring in different settings) and they must lead to significant problems (at least moderate impairment from symptoms in two or more domains, e.g., education, work, relationships).

In addition to a detailed exploration of core ADHD symptoms and functional impairments, it is essential to screen for other mental health and neurodevelopmental disorders and evaluate whether the presenting symptoms may be better explained by another condition. This requires a good understanding both of ADHD and overlapping conditions.

If an individual is likely to be offered medication for their ADHD, a careful review of the medical (and family medical) history is required to ensure there are no contraindications or cautions with respect to ADHD medication management.

Finally, if the diagnosis of ADHD is made, it is important to have what NICE refers to as a ‘post-diagnostic discussion’ (16). Here you can start to link the diagnostic outcome with the beginnings of a treatment plan that not only considers medication, but also sensitively addresses psychosocial, educational, and vocational issues. Although it isn’t necessary to focus on the specifics of the management plan in the same session as the assessment, the management journey very much starts there. Ideally, at the very least, some time would be dedicated to overviewing management options, if only to seed ideas for personal research between sessions. A subsequent session would usually be arranged to discuss management in more detail.

#### 3.1.1 Consensus statements on adult ADHD diagnostic assessments

##### 3.1.1.1 Pre-assessment self-completed baseline data collection

1. Which self-completed pre-assessment (baseline) scales are used and what other data is collected prior to the session is up to the individual clinician, but the focus should be on the patient’s presenting problems, what they want help with and the reason for requesting a diagnostic assessment. Self-completed rating scales (e.g., 18-point ADHD symptom scales) should only be used to provide a baseline for the subsequent evaluation of change following diagnosis and treatment. They should not serve as a formative part of the diagnostic assessment as they typically consist of a list of leading questions that follow a closed-question format (i.e., yes or no answers, or Likert scale). Their potential role to screen for ADHD without clinical assessment is not currently supported (55). A useful option for measuring functional change following treatment are scales that measure impairment in different aspects of daily life (e.g., the Weiss Functional Impairment Rating Scale) [optional].
2. Validated questionnaires for co-morbidities may also be included to screen for other mental and physical health disorders (e.g., a screening tool for Autistic Spectrum Disorder such as the AQ-10, or scales for anxiety and depression) [optional].
3. Various forms of qualitative data may be collected e.g., the patient may be asked to write their life story, or produce a timeline [optional].

##### 3.1.1.2 The assessment itself (direct clinical contact)

1. In the case of an assessment prior to initiating medication, it is also important to elicit the relevant information required to demonstrate that there are no obvious barriers to prescribing, including details about comorbidities, cardiovascular status, substance misuse and other risk issues [Essential].
2. Orientation to assessment process: Providing an overview of the assessment process, either in advance or at the start of the session, is important in setting expectations for patients [highly recommended].
3. Information gathering: All relevant information obtained during the assessment needs to be captured and noted in sufficient detail [Highly recommended].

##### 3.1.1.3 Systematic evaluation of ADHD symptoms

1. Each of the DSM-5 ADHD symptoms and additional diagnostic criteria need to be carefully evaluated using a systematic approach. It is essential to elicit and document clear examples of each of the symptoms identified as being present [essential].
2. This should be done using a semi-structured interview assessment to ensure each item is addressed [essential].
3. Recommended interview schedules include DIVA, CAADID or ACE+. Alternatively, an experienced ADHD assessor may follow an interview style adapted to individual patients, ensuring that all DSM symptoms, impairments and additional criteria are fully explored [Essential].
4. This assessment should always be conducted via direct consultation (face-to-face, or remote using video-conferencing). This is essential as the patient needs to be probed by the assessor to elicit their subjective experiences and behaviours in real life situations [essential].
5. Open-ended questions should be used to avoid leading the patient [essential].

##### 3.1.1.4 Structured assessment of impairment

1. Evaluating impairment in functioning from the symptoms of ADHD, is a core component of the ADHD diagnosis [essential].
2. Employment and educational history may be particularly revealing. The interview should enquire about employment and education related examples in which core ADHD symptoms have interfered with or reduced the quality of functioning in these settings [essential].
3. Function in social and home settings is equally important and should be explored in all cases. This can be more impairing than occupational function in some cases [essential].
4. It is recommended to capture the detail under subheadings such as education, employment, leisure, family/close relationships, friendships, daily tasks and self-image. [highly recommended].

##### 3.1.1.5 Developmental history

A careful and systematic review of the individual’s life history, spanning childhood, adolescence, and adulthood should be completed, and may include:

**General considerations**

1. Onset of ADHD symptoms and impairments: The presence of symptoms and impairments directly attributable to ADHD must not simply be based on a ‘snapshot’ clinical impression gained during the consultation. See below for guidance on the use of semi-structured clinical interviews [essential].
2. Other neurodevelopmental disorders: Screen for other neurodevelopmental disorders such as autism, tics and Tourette’s, dyslexia and dyspraxia, and other learning disabilities including intellectual disability [essential].
3. 3 Self-completed life-story or time-line: It may be helpful to ask the patient to provide a (one page) written ‘life story’ or timeline to capture additional information [optional].

**Psychosocial development and early risk factors**

1. Childhood and adolescence: academic progress, exclusions/expulsions, behaviour in class, peer group relationships, antisocial behaviour, personal relationships and traumatic experiences [essential].
2. Adult life: Educational and employment record, social and family/personal life [essential].
3. Pregnancy-related details [highly recommended].
4. Birth and infancy: birth complications, premature birth, low birth weight, developmental milestones, early temperament [highly recommended].

##### 3.1.1.6 Mental health history

1. Adulthood: It is essential to screen for common mental health problems (both current and previous), particularly those that can mimic ADHD, or are commonly comorbid with ADHD. It is also important to enquire about the presence of disorders (e.g., binge eating, anorexia or bipolar disorder) that may change the management of ADHD if diagnosed [essential].
2. Health-care records: If patients have had previous contact with mental health services, it is good practice to review (electronic) care records, including GP letters, previous assessment and medico-legal reports. This can be time-consuming but is essential for more complex patients. [highly recommended].

##### 3.1.1.7 Medical and physical health history

1. Before medication is considered, it is necessary to carry out an assessment of cardiovascular status (16). This should include screening for current or previous cardiovascular or blood pressure-related problems (including orthostatic symptoms i.e., symptoms on standing), and a family history of cardiovascular problems (see below). An electrocardiogram (ECG) is not routinely required. Always record baseline blood pressure, pulse and weight [essential].
2. Comorbidities: Screen for common comorbidities that include obesity, allergies and atopy (including asthma), hypermobility, chronic fatigue syndrome, fibromyalgia, dysautonomia (e.g., POTS), diabetes, migraine, irritable bowel syndrome, glaucoma, thyroid issues and autoimmune conditions such as psoriasis and ulcerative colitis [highly recommended].
3. Explore current health status, including sleep and diet [highly recommended].
4. Ask specifically about a history of seizures or head injuries [highly recommended].

##### 3.1.1.8 Personal and family substance use, addiction and forensic history

1. Risk (or ‘safety’) assessments should always be completed, particularly considering risks posed by other mental health issues, substance abuse, and the potential for misuse/diversion of prescribed medications [essential].
2. ADHD is an established risk factor for alcohol and substance misuse, and if present this can impact management, so this is a particularly important area to explore [essential].
3. Substance use, addiction and forensic history should be approached in the same manner as for any other mental health disorder, as described in standard mental health textbooks (56) [highly recommended].

##### 3.1.1.9 Informant perspective(s)

1. Whenever possible information should be gathered from one or more informants [highly recommended].
2. Childhood history: Ideally, at least one of the informants should be someone who knew the individual during their childhood years (usually a parent or older sibling). They will be able to contribute to the developmental history, and give a perspective on age of onset and the impact of the ADHD symptoms during early years [highly recommended].
3. Current functioning: It is also important to get an informant account from someone who has known the patient well during their adult years, to get a perspective on current, day-to-day functioning [highly recommended].
4. Informants related to childhood and adulthood may be the same person, but often they are not. Due to the practicalities and time considerations of having multiple informants attending assessments, requesting a written summary via the patient pre-assessment (with clear instructions provided, indicating what you want to know) from one or more of the informants can be a helpful alternative. Their narrative (or extracts from it) can be included verbatim in the assessment report, if appropriate [optional].
5. Informant completed rating scales are sometimes used as an alternative where interview assessments are not practical [optional].
6. Joint assessments: Where there is a close relationship between an informant and the patient (e.g., a supportive parent, sibling and/or partner), and if the patient consents and doesn’t feel inhibited a joint interview for part or the full assessment can be conducted. This can be helpful and may improve the efficiency of information gathering [optional].

##### 3.1.1.10 Post-diagnostic structured discussion

1. According to NICE, following a diagnosis of ADHD (and prior to discussing treatment), there must be evidence of a structured discussion with patients (and their families or carers as appropriate) about how the diagnosis of ADHD may affect their life. This discussion may take place at the end of the assessment but can be deferred to a subsequent session dedicated to management [essential].
2. The discussion should always address environmental modifications to reduce the impact of ADHD symptoms (16). Environmental modifications refers to strategies to reduce the impact of ADHD symptoms by, for example, alterations to work tasks or the working environment, or learning strategies to help with organisation or task initiation and completion [essential].
3. The discussion should ideally include the positive impacts of receiving a diagnosis, that have been covered in a number of papers (57). [highly recommended].
4. It should also explore the potential negative impacts of receiving a diagnosis, such as stigma and labelling, and the fact that ADHD can result in impulsive and potentially risky behaviour [optional].

**Other areas to discuss include:**

1. Education and employment issues [highly recommended].
2. Social and relationship issues [highly recommended].
3. The increased risk of substance misuse and self-medication [highly recommended].
4. The potential impact on driving (see Theme 2 below for further detail related to ADHD and driving) [highly recommended].
5. Pharmacological and psychosocial treatment options should be explored (perhaps just broadly overviewed initially) [highly recommended].
6. The challenges of managing ADHD in the presence of coexisting neurodevelopmental or mental health conditions [optional].
7. Signposting/referral to NHS services, or third sector support organisations as required [optional].
8. The importance of good long-term self-care and lifestyle management, nurturing a ‘growth mindset’ and fostering more acceptance and self-compassion, will undoubtedly serve to provide a cornerstone for future engagement and more effective self-management [optional].

### 3.2 Theme 2: What information should be included in adult ADHD assessment reports?

Assessment reports should, as far as possible, provide evidence of having covered the defined assessment domains (outline in Theme 1 above). Where ‘essential’ information is not available, it should be made clear in the assessment report that it was discussed and/or considered.

#### 3.2.1 Consensus statements on adult ADHD assessment reports

##### 3.2.1.1 General considerations

1. All relevant information obtained during the assessment needs to be recorded in sufficient detail in the assessment report to demonstrate a robust and valid diagnostic assessment. The diagnostic conclusions reached must be comprehensively supported by the information included in the assessment report [essential].
2. To fulfil diagnostic criteria, the assessment report needs to confirm at some point that ‘the symptoms are not better explained by another disorder’, which is a distinct criterion [essential].
3. It is important that the assessment headings outlined in Theme 1 are broadly covered in the assessment report. In general, it is not appropriate to omit headings or to populate them with insufficient, irrelevant, or repetitious information from other sections [highly recommended].
4. Where there is missing information, or evidence that seems to contradict the diagnostic conclusions, there should be an exploration of the issue and its impact on the validity of the conclusion. Assessing clinicians should give careful thought as to whether they can justify making a diagnosis of ADHD, in the context of missing or contradictory information [highly recommended].
5. It is acknowledged that the length of a report does not necessarily serve as a marker for quality. Nevertheless, the relevant information recommended by the AQAS needs to be summarised in sufficient detail. It is not sufficient to simply bulk out the report with generic tables, graphs, appendices, references, information relating to the assessor or service, or recommendations for treatment) [highly recommended].

##### 3.2.1.2 Key components of the adult ADHD assessment report

1. An appropriately detailed account of the specific symptoms identified - see section above for further detail [essential].
2. The accompanying narrative should include real-life examples of symptoms and, where relevant, note the context in which they occur [highly recommended].
3. Scores from semi-structured interviews (as well as the scores from the other self-report and informant questionnaires used, if applicable) with an accompanying narrative of sufficient detail [highly recommended].
4. Clear evidence that informant information was sought (see Theme 1 for approach), and a brief explanation of how this has helped build the clinical picture and inform the diagnostic formulation [highly recommended].
5. An indication that school reports (and other relevant assessment reports) have been reviewed, where available, and whether they are supportive of the diagnosis [highly recommended].
6. Informant narrative (or extracts from it) can be included verbatim in the assessment report, if appropriate [optional]
7. Verbatim excerpts (or otherwise a summary of themes) from reports can be included to illustrate specific points [optional].
8. A summary of previous and current mental and physical health comorbidities [essential].
  - Consideration should be given as to how the comorbid condition may be impacting or contributing to the ADHD presentation (or vice versa), as in some cases the comorbidity may need to be addressed first [highly recommended].
  - It is important to comment in the assessment report on the absence of certain relevant conditions (e.g., “no evidence of previous head injuries or seizures; no evidence of blood pressure or cardiovascular problems past or present; no history of joint hypermobility/orthostatic symptoms/allergy etc”) [highly recommended].
9. Clear documentation of a formulation and the diagnostic outcome (i.e., ADHD diagnosis confirmed or not) including clear evidence that diagnostic criteria have been reviewed and met or not met, as appropriate [essential].
  - For DSM-5-TR, and specifically in the case of adults, this entails demonstrating that 5 or more symptoms were identified in either the inattentive and/or hyperactive-impulsive symptom domains (Criteria A), as well as the additional criteria (B-E) required for the diagnosis [essential].
  - If ADHD diagnostic criteria are met, the symptom scores from the two symptom domains guides the clinical presentation subtype, that should be explicitly stated in the report (e.g., ADHD–combined presentation, ADHD–predominantly inattentive presentation, ADHD–predominantly hyperactive-impulsive presentation) [essential].
  - Where ADHD diagnostic criteria are not met, the report should clarify whether any ADHD symptoms are arising in the context of a subthreshold diagnosis of ADHD (for example symptoms and impairments may diminish with age, or patients may fall just below diagnostic levels of symptoms or impairments); or may be secondary to an different mental, medical or neurodevelopmental condition [highly recommended].
10. A consideration of driving safety should be considered. If a diagnosis is made (and the patient presently drives or is learning to drive) they should be advised to look at the Driver and Vehicle Licensing Agency (DVLA, UK) criteria and guidance (that can change over time). Ideally, this should be mentioned in the assessment report. If there are significant driving safety concerns, these would be raised with the patient, and in the report, including the detail of the action taken [highly recommended].

### 3.3 Theme 3: What levels of impairment are required to make a diagnosis of adult ADHD?

Adult ADHD presents with a wide range of impairments, impacting multiple areas of life. The impairment can be directly linked to ADHD, but may in part also be explained by any accompanying comorbidities, including substance use disorder, and anxiety and depressive disorders, amongst others.

Symptoms of ADHD (inattention, hyperactivity and impulsivity) are on a continuum in the general population, and are present to some extent in the non-ADHD population (6), with most people being able to relate to some of the features of ADHD to some degree. Therefore, it is important to determine not only the presence of the symptoms, but also evaluate their severity and the level of associated functional impairment. This is one of the key skills of the assessor.

The assessment of impairment can be supported by the use of scales such as the Weiss Functional Impairment Rating Scales (58) and the WHO Disability Assessment Schedule (59), but it should be primarily underpinned and informed by information gathered during the assessment interview.

Importantly, diagnosis of ADHD should not be ruled out simply on the basis of the presence of high levels of achievement in certain domains of life (e.g., in academic or professional settings). The individual may well be underachieving relative to their potential, and there may well be other areas of impairment that may not be immediately apparent. These may include impairment in social or family functioning, sleep problems, ADHD-related emotional dysregulation, or internalising symptoms related to ADHD such as exhausting levels of mental and physical restlessness, sustained low self-esteem, fatigue, or high stress levels. Such individuals are sometimes referred to as having “high-functioning” adult ADHD; a term that may obscure considerable illness burden.

#### 3.3.1 Consensus statements on the assessment of impairment

1. DSM-5-TR criteria for ADHD states that a diagnosis of ADHD can only be made if the symptoms interfere with or reduce the quality of social, academic, or occupational functioning. Following guidance from NICE, impairment from the symptoms of ADHD should be at least of moderate severity and in at least two domains or settings (including psychological functioning), based on interview and/or direct observation, [essential].
2. The assessment report should indicate the domains in which there is at least moderate impairment [essential].
3. As introduced in Theme 1, when assessing for impairment, it can be helpful to consider the following domains: education, employment, leisure/social, family/close relationships, friendships, daily-tasks and self-image [highly recommended].

### 3.4 Theme 4: How long should an adult ADHD diagnostic assessment take?

The NICE Guideline for ADHD provides some limited guidance on the duration of ADHD assessments (14, 16). It stipulates that clinicians should ensure that there is sufficient time for the patient to describe and discuss their problems, for the clinician to summarise the conclusions of the assessment, and for discussion. It mentions that additional time may be needed for a more extensive discussion with those who are dissatisfied with the outcome of the assessment. The guideline also recommends that patients are made aware of how long the assessment will take in advance, so that they allow enough time.

The Royal College of Psychiatrists in Scotland good practice guidelines considers that “in most cases, assessment and diagnosis of ADHD will require two to three 1h sessions”. It adds “It is important to stress that this process does not need to be rushed; by definition, these patients are likely to have experienced these difficulties for many years. For some, evidence for the diagnosis of ADHD may be fairly compelling by the end of the first 1h appointment. For others, without clear evidence of the disorder, the process will end at this stage or may proceed to consider a different diagnosis” (18).

Amongst the co-authors there was a clear consensus that a thorough assessment is absolutely necessary for an accurate and valid diagnosis, and to create solid foundations for the successful longer-term management of this often lifelong condition. There was also a clear acknowledgement that to cover what is required in a meaningful way takes a reasonable amount of time, however efficient the clinician is.

The amount of time that should be allocated to assessment was extensively debated, and a consensus was reached regarding the recommendations. There was, throughout the discussions, an acute awareness of resource limitations and the considerable unmet need, running alongside a shared conviction that things need to be done properly, and to a required standard. Allowing standards to slip, as they have in some cases, will ultimately disadvantage the patient.

#### 3.4.1 Consensus statements on the duration of adult ADHD diagnostic assessments

To complete an adequate assessment for adult ADHD there needs to be sufficient time for the following:

- to take an adequate developmental history.
- to obtain a full psychiatric/personal/family/relationship/work/forensic/alcohol and substance use history, and carry out a risk assessment.
- to elicit both a current and retrospective account of ADHD symptoms and impairments.
- to screen for associated co-morbidities.
- to discuss the assessment outcome, allowing time for the patient to reflect and ask questions.
- to provide some initial psychoeducation.
- to overview treatment options.

This is expected to take two hours or more of direct patient contact time, as most experienced clinicians report that adult ADHD assessments typically take them between 2 and 3 hours.

The duration of the assessment is not however an absolute, as it is very dependent on the complexity of the case, and in particular, the comorbidities present and psychiatric history. An additional factor is the experience of the clinician. The assessment may take longer for less experienced clinicians, compared to those with more extensive experience of evaluating ADHD.

We therefore recommend the following:

1. Regardless of the approach taken, the total assessment time should be at least 2 hours in most cases. This may take the form of one 2–3-hour consultation (often with a break), or a number of shorter consultations, totalling at least two hours in most cases [essential].
2. Some services split the assessment process into an initial diagnostic interview for ADHD (around 90 minutes, including a semi-structured interview), with a subsequent session covering the other components, including detailed feedback, time for patients to reflect on the outcome and a discussion about the range of treatment options (usually another hour) [optional].
3. Some services may offer a multi-disciplinary assessment that can work well if the process is well coordinated, with the required oversight and communication [optional].
4. Additional time may be required if more junior or less experienced practitioners need to discuss the case with a more experienced practitioner (e.g., a psychiatrist or psychologist who has not been involved directly) [optional]

### 3.5 Theme 5: What core competencies are required to conduct a diagnostic assessment of adult ADHD?

An accurate diagnosis of adult ADHD requires a skilled and knowledgeable practitioner. One of the necessary skills is knowing when not to make a diagnosis, something that requires a sound understanding of both ADHD, and other mental, neurodevelopmental, and sometimes physical health conditions.

In line with the general approach to all mental health conditions, the diagnosis of adult ADHD is dependent on clinical judgement as to the presence or absence of certain symptoms, and whether a required threshold for impairment has been reached. It is therefore critically important to capture the necessary data during the assessment process, to ensure an adequately informed clinical judgement by an adequately trained and experienced clinician.

The amount of training that a clinician requires to work with adult ADHD currently remains somewhat vague in existing practice guidelines. Surveys conducted in the United States (60) and Germany (54), have demonstrated that many clinicians feel uncertain about their ability to diagnose adult ADHD accurately. Additionally, a survey by the Royal College of Psychiatrists reported that 32% of psychiatrists were not confident in assessing neurodevelopmental disorders, including ADHD, and 67% reported that they wanted further training (23).

When assessing an individual for adult ADHD, a clinician is expected to be able to consider a range of differential diagnoses. To do that, they need to be familiar with, and competent in assessing a range of mental health disorders, as well as physical health conditions. There is an increasing appreciation of ADHD’s physical health comorbidities.

Additionally, the clinician will need to understand the medical factors that may influence how safe it is to prescribe adult ADHD medications, how an in-depth evaluation of the patient’s family history may guide decision-making, and what precautions and monitoring may be necessary.

It was broadly agreed that to gain this level of experience and knowledge, at a minimum, one needs to have been working clinically in the mental health field for several years. Ideally clinicians will have trained in ADHD alongside their training in other common mental health and neurodevelopmental disorders. This should minimise both false positive and false negative ADHD diagnoses. Developing a broader perspective in this area is essential, as ADHD is one of many common conditions impacting on mental health and functioning in everyday life (11).

There is growing acceptance and agreement that there should also exist ‘non-medical’ assessments for adult ADHD, that are typically carried out in higher educational settings (61). Such assessments can expedite the assessment process, more rapidly establish if the diagnosis of adult ADHD is likely and initiate early and appropriate support. These assessments are particularly focused on how the presenting symptoms are impacting on educational functioning and attainment. This is necessary, in part, to justify and guide the student’s requirements for adjustments during this critical period of education. With the appropriate training these assessments can be of exceptionally high quality, identifying ADHD characteristics, leading directly to non-medical forms of support in a timely manner. For example, such assessments are used to access student disability allowances, and rapid access to educational support for learning difficulties related to ADHD. They may also be especially helpful if the student would like to explore the option of trying ADHD medication, potentially allowing a ‘fast-tracking’ to a briefer medical assessment by mental health services. This may be facilitated by good report writing and communication between non-medical and medical assessors.

#### 3.5.1 Consensus statements on assessor competencies

1. In accordance with NICE guidelines for ADHD (16), a diagnosis of ADHD should only be made by “a specialist psychiatrist, paediatrician or other appropriately qualified healthcare professional with training and expertise in the diagnosis of ADHD”. The guideline stipulates that “they should undertake training so that they are able to diagnose ADHD and provide treatment and management in accordance with this guideline”. [essential].
2. Healthcare professionals assessing for adult ADHD need to have been working clinically in the mental health field for several years. This may include those working in another primary role (e.g. neurology, primary care) with extensive experience of mental health [highly recommended].
3. Those assessing ADHD need to have received specific teaching/training on the assessment, and ideally the treatment, of adult ADHD. This training needs to span sufficient hours as part of a formal course, rather than simply involving attending a brief teaching session [highly recommended].
4. Following their initial training, clinicians will need to arrange supervision for their adult ADHD cases. Supervision should be provided by someone, ideally within their local network, with extensive experience of adult mental health and the assessment and treatment of ADHD (see section 6.1).
5. The number of cases that one needs to have supervised to integrate the necessary learning depends on demonstrating acquisition of the skills required, and should be kept under review. The consensus among the co-authors is that it should be between 10 and 20. Good practice would involve these supervision sessions being documented and for the supervision to be captured and reflected on as part of the clinician’s appraisal [highly recommended].

#### 3.5.2 Which professions can diagnose ADHD?

Consultant psychiatrists and health care professionals with extensive experience of adult mental health have the basic skills required. Clinicians in other professional areas may also acquire the specialist skills and competencies required to be an assessor of adult ADHD.

1. The specialities that generally have the necessary experience and expertise to assess and manage adult ADHD without frequent supervision, following training in ADHD, include the following (note: this list is not exhaustive): consultant psychiatrists, associate specialists, consultant clinical psychologists, advanced nurse practioners (Band 8a, with non-medical prescribing training) and nurse consultants (Band 8B and 8C), and other health care professionals with extensive experience in mental health.
2. Specialities that will typically require ongoing formal supervision (at least monthly), include the following: psychiatric trainees at speciality trainee (ST) level, mental health nurses at Band 6/7 level (including non-medical prescribers), clinical psychologists at Band 7/8A/8B level, and GPs developing a special interest or extended role working in ADHD services.
3. Other professionals who can assist in the assessment process (with specialist oversight) include: GPs with an interest in learning more about adult ADHD, junior mental health nurses, junior psychiatric trainees, assistant psychologists, educational psychologists, and associate mental health workers
4. Professionals who may be involved in screening for adult ADHD and making onward referrals include: psychiatrists (all levels), doctors from any medical discipline where ADHD is commonly seen (e.g. rheumatologists, neurologists etc), GPs and other clinicians in primary care, clinical psychologists psychologisgts and educational psychologists and assessors.

## 4 Discussion

The recent increase in demand for adult ADHD assessments has exposed considerable heterogeneity in quality. In 2020, prior to the COVID-19 pandemic, freedom of information requests obtained by the British Broadcasting Corporation (BBC) indicated that at least 21,000 adults in the UK were on a waiting list for an ADHD assessment, with many people waiting 2-5 years for an appointment (62). This very high level of unmet need has in our view led to a dilution of standards in some clinical services that has impacted quality of care, particularly for adults with complex presentations of ADHD.

In response to the need to maintain good standards of care, UKAAN developed the AQAS. The resulting recommendations meet the overarching aim of UKAAN to support services in providing adults with ADHD with high quality assessments, carried out by suitably trained and experienced professionals, with access to suitable peer group or individual supervision. The goal is to ensure that adults with ADHD are recognised and accurately diagnosed, and then go on to receive optimal, evidence-based treatments for this common and impairing but treatable condition.

The development of the AQAS is designed to support this process. It achieves these aims by providing clear, practical and sufficiently detailed guidance for the assessment of adult ADHD. The routine use of the AQAS by adult ADHD assessors is intended to promote high quality assessments, improve the accuracy of ADHD diagnoses and reduce the risk of overdiagnosis, misdiagnosis and underdiagnosis. Following the AQAS recommendations for the writing of reports should facilitate more effective communication and liaison between adult ADHD specialists/specialist teams and other services, including primary care and those in the education sector. The AQAS also supports patients and other service users by helping them to understand what to expect from the assessment process.

The AQAS is not intended to replace formal training and supervision but rather to provide a useful framework for those who have done the necessary training to follow. The aim is not to be overly prescriptive, but to provide sensible, and well considered recommendations to support high quality clinical care.

One concern that surfaced several times and was hotly debated during the consensus process, is the recommended minimum assessment time of 2 hours. Whilst we recognise this may be challenging for some services to deliver, we consider it necessary to ensure both the accuracy of assessments and the optimal long-term care of individual patients. As discussed, assessments can be broken down into two or more sessions if required, and we do not discourage (high quality) multidisciplinary assessments, both of which should provide a degree of flexibility for adult ADHD service providers. Our recommendations in this area are in line with the Scottish Royal College of Psychiatrists recommendations (18).

We acknowledge that more straightforward cases will need a shorter assessment time than more complex cases, but not at the expense of a basic quality threshold. Inappropriately short and inadequate assessments are far more likely to result in diagnostic errors and a resultant negative impact on patient care. This can include unnecessary distress for patients who receive incorrect diagnoses and are then exposed to costly, unnecessary, and potentially harmful medical and psychosocial interventions.

## 5 Key recommendations

The AQAS has been developed to enhance the patient experience, and the delivery of optimal care for adult ADHD, in line with the UK NICE guidelines. A summary of AQAS procedures and the items needed for the diagnostic assessment and post-diagnostic discussions are listed in **Tables 1**–**3**. Key recommendations for how AQAS should be conducted are as follows:

1. Assessment of adult ADHD should not be seen in isolation but as only one component of a full psychiatric and neurodevelopmental review.
2. The assessor must keep an open mind in terms of the diagnostic landscape - ADHD may be one of several potential co-existing diagnoses contributing to the impairment burden experienced over the patient’s lifespan. The high rates of comorbidity with ADHD can make this a considerable task.
3. ADHD should not be diagnosed when another condition better explains the presenting symptoms and impairments.
4. The assessor must be familiar with autism spectrum, mood/bipolar, personality and substance use disorders, and other comorbidities and differential diagnoses. It is also important to consider physical comorbidity, particularly conditions that have a bearing on prescribing medication for ADHD or may cause symptoms resembling those of ADHD.
5. To conduct adequate assessments, assessors need to have good interview skills. A semi-structured interview must allow open questions with appropriate elaboration and reflection. Probing is required to elicit real life examples of symptoms and impairments in the individual’s daily life. Assessments that rely on a checklist of closed questions (“tick boxes”) based on the 18 ADHD symptoms of inattention, hyperactivity and impulsivity run the risk of confirmation bias, and may fail to properly consider important differential diagnoses. Relying excessively on rating scales and pre-test questionnaires for evidence of symptoms may play into this.
6. The direct contact time of the diagnostic assessment is likely to be at least 2 hours in most cases, conducted over single or multiple sessions; including the assessment itself, discussion of the diagnosis and treatment and initial psychoeducation. Timing may be longer depending on the complexity of the case, and the skill of the assessor.
7. Objective or third-party information, where possible, should be used to corroborate subjective accounts and elucidate the nature of symptoms and severity of impairment.
8. The report should give examples that illustrate how the diagnostic criteria are met and provide sufficient description of the symptoms and impairments, to allow other stakeholders to have confidence in the diagnosis.
9. After diagnosis, it is important to provide the patient with detailed explanation and psychoeducation about ADHD, in understandable language. The assessor must allow the patient to reflect on the diagnosis and the opportunity to ask follow-up questions. Psychosocial issues should be discussed, including educational, occupational, and social impacts (including driving).
10. The complexity of the cases referred for assessment can vary, dependent on the nature of referral pathways, including self-referral, thresholds for referral, and how cases are screened prior to formal assessment. Information obtained from referral letters, previous medical notes, questionnaires etc. can be an important part of the assessment.

**Table 1:** Summary list of AQAS procedures.

▫ Pre–Assessment (baseline) information ▫ A detailed account of presenting issues suggestive of ADHD (covering both childhood and adulthood) ▫ Developmental history (which may include a patient–written ‘life story’ or timeline) ▫ A review of school (or other relevant) reports ▫ Informant input (i.e., partner, parent, friend) of both a qualitative and quantitative nature ▫ Use of a semi–structured diagnostic interview ▫ A consideration of impairment ▫ Screening for mental and physical health comorbidity (including other neurodevelopmental conditions and conditions that are commonly associated with ADHD) ▫ An assessment of cardiovascular status (including baseline blood pressure, pulse and weight) prior to initiating medication ▫ A screen for risk/safety issues ▫ A cross reference to the diagnostic criteria ▫ A discussion about treatment options (and other relevant information)

**Table 2:** Essential components for diagnostic assessment.

▫ Presenting complaint(s). ▫ Full psychiatric history. ▫ Neurodevelopmental evaluation. ▫ Past medical history for potential differentials and contraindications to treatment. ▫ Semi-structured interview with open questions and elaboration; not merely a checklist; not simply “yes or no” questions; careful use of, not reliance on, questionnaires or rating scales- with adequate probing during the interview. All relevant areas of life (e.g., education, employment, leisure, relationships, daily task and health management) need to be reviewed in detail to establish the impact of symptoms and the degree of impairment. ▫ Explicit detailing of which DSM-5 symptoms of inattention, hyperactivity and impulsivity are present, pervasive, and persistent; and how they cause at least moderate impairment in at least two domains, with detailed illustrative examples. ▫ Discussion of independent evidence (informant-based and other objective data such as school reports) used to support the diagnosis. ▫ Consideration of differential diagnoses & comorbidity (including physical health comorbidity).

**Table 3:** Essential components for post–diagnostic discussion and procedures.

▫ Detailed feedback, explanation. and psychoeducation about ADHD, in clear language. ▫ A discussion about psychosocial issues, including education or occupation and driving. ▫ Time for the patient to reflect on the diagnosis and ask questions. ▫ A discussion to facilitate shared decision making about available treatment options, consideration of contraindications to prescribing certain medication (if relevant), and reasons for preferring one treatment to others. ▫ Consideration of measurable treatment goals before starting treatment. ▫ Physical monitoring for medication (clinical examination, blood pressure, pulse and weight) at baseline and during treatment. ▫ Seeking agreement from the GP for future prescribing, recognising different approaches to ‘shared care’.

## Author contributions

MAd: Conceptualization, Writing – original draft, Writing – review & editing. MAr: Conceptualization, Writing – original draft, Writing – review & editing. PA: Conceptualization, Writing – original draft, Writing – review & editing. SC: Conceptualization, Writing – original draft, Writing – review & editing. LL: Conceptualization, Writing – original draft, Writing – review & editing. JS: Conceptualization, Writing – original draft, Writing – review & editing. UM: Conceptualization, Writing – original draft, Writing – review & editing. KvR: Conceptualization, Writing – original draft, Writing – review & editing. JK: Conceptualization, Writing – original draft, Writing – review & editing.

## References

[1] Fayyad J Sampson NA Hwang I Adamowski T Aguilar-Gaxiola S Al-Hamzawi A. Florescu S et al: The descriptive epidemiology of DSM-IV Adult ADHD in the World Health Organization World Mental Health Surveys. Atten Defic Hyperact Disord. (2017) 9:47–65. doi: 10.1007/s12402-016-0208-3 27866355 PMC5325787

[2] Schoeman R Albertyn R de Klerk M. Adult attention-deficit hyperactivity disorder: Why should we pay attention? S Afr J Psychiatr. (2017) 23:a1072. doi: 10.4102/sajpsychiatry.v23i0.1072 PMC6138203 30263197

[3] Moncrieff J Timimi S. Is ADHD a valid diagnosis in adults? No. Bmj. (2010) 340. doi: 10.1136/bmj.c547 20348183

[4] Kooij SJ Bejerot S Blackwell A Caci H Casas-Brugué M Carpentier PJ. European consensus statement on diagnosis and treatment of adult ADHD: The European Network Adult ADHD. BMC Psychiatry. (2010) 10:1–24. doi: 10.1186/1471-244X-10-67 20815868 PMC2942810

[5] Franke B Michelini G Asherson P Banaschewski T Bilbow A Buitelaar JK. Live fast, die young? A review on the developmental trajectories of ADHD across the lifespan. Eur Neuropsychopharmacol. (2018) 28:1059–88. doi: 10.1016/j.euroneuro.2018.08.001 PMC6379245 30195575

[6] Asherson P Buitelaar J Faraone SV Rohde LA. Adult attention-deficit hyperactivity disorder: key conceptual issues. Lancet Psychiatry. (2016) 3:568–78. doi: 10.1016/S2215-0366(16)30032-3 27183901

[7] Faraone SV Banaschewski T Coghill D Zheng Y Biederman J Bellgrove MA. The world federation of ADHD international consensus statement: 208 evidence-based conclusions about the disorder. Neurosci Biobehav Rev. (2021) 128:789–818. doi: 10.1016/j.neubiorev.2021.01.022 PMC8328933 33549739

[8] Frances A FM Pincus HA. DSM-IV guidebook. Washington DC: American Psychiatric Press (1995).

[9] Epstein JN Loren RE. Changes in the definition of ADHD in DSM-5: subtle but important. Neuropsychiatry. (2013) 3:455. doi: 10.2217/npy.13.59 24644516 PMC3955126

[10] Adamou M Graham K MacKeith J Burns S Emerson LM. Advancing services for adult ADHD: the development of the ADHD Star as a framework for multidisciplinary interventions. BMC Health Serv Res. (2016) 16:632. doi: 10.1186/s12913-016-1894-4 27821125 PMC5100092

[11] Asherson P Leaver L Adamou M Arif M Askey G Butler M. Mainstreaming adult ADHD into primary care in the UK: guidance, practice, and best practice recommendations. BMC Psychiatry. (2022) 22:640. doi: 10.1186/s12888-022-04290-7 36221085 PMC9553294

[12] Bolea-Alamanac B Nutt DJ Adamou M Asherson P Bazire S Coghill D. Evidence-based guidelines for the pharmacological management of attention deficit hyperactivity disorder: update on recommendations from the British Association for Psychopharmacology. J Psychopharmacol. (2014) 28:179–203. doi: 10.1177/0269881113519509 24526134

[13] Smith MCF Mukherjee RAS Muller-Sedgwick U Hank D Carpenter P Adamou M. UK adult ADHD services in crisis. BJPsych Bull. (2023) 2023:1–5. doi: 10.1192/bjb.2023.88 PMC10801359 38058161

[14] National Institute for Health and Care Excellence. Attention Deficit Hyperactivity Disorder: Diagnosis and management of ADHD in children, young people and adults. Leicester, UK: British Psychological Society (2009). 22420012

[15] Taylor E Asherson P Bretherton KV Hollis C Keen D Ryan N. Attention deficit hyperactivity disorder. Evidence update 45. Manchester, UK: National Institute of Health Care Excellence.

[16] National Institute of Health and Care Excellence. Diagnosis and management of ADHD in children, young people and adults. In: London The British Psychological Society and The Royal College of Psychiatrists (2018). Available online at: https://www.nice.org.uk/guidance/ng87/resources/attention-deficit-hyperactivity-disorder-diagnosis-and-management-pdf-1837699732933.

[17] Nutt DJ Fone K Asherson P Bramble D Hill P Matthews K. Evidence-based guidelines for management of attention-deficit/hyperactivity disorder in adolescents in transition to adult services and in adults: recommendations from the British Association for Psychopharmacology. J Psychopharmacol. (2007) 21:10–41. doi: 10.1177/0269881106073219 17092962

[18] ADHD in adults: good practice guidelines . Available online at: https://www.rcpsych.ac.uk/docs/default-source/members/divisions/scotland/adhd_in_adultsfinal_guidelines_june2017.pdf.

[19] Sibley MH. Empirically–informed guidelines for first–time adult ADHD diagnosis. J Clin Exp Neuropsychol. (2021), 1–12. doi: 10.1080/13803395.2021.1923665 33949916

[20] Young S Asherson P Lloyd T Absoud M Arif M Colley WA. Failure of healthcare provision for attention–Deficit/Hyperactivity disorder in the United Kingdom: A consensus statement. Front Psychiatry. (2021) 12:649399. doi: 10.3389/fpsyt.2021.649399 33815178 PMC8017218

[21] Retz W Retz–Junginger P Thome J Rosler M. Pharmacological treatment of adult ADHD in Europe. World J Biol Psychiatry. (2011) 12 Suppl 1:89–94. doi: 10.3109/15622975.2010.540257 21906003

[22] Tachmazidis I Chen T Adamou M Antoniou G. A hybrid AI approach for supporting clinical diagnosis of attention deficit hyperactivity disorder (ADHD) in adults. Health Inf Sci Syst. (2021) 9:1–8. doi: 10.1007/s13755-020-00123-7 33235709 PMC7680466

[23] Royal College of Psychiatry. A competency based curriculum for specialist training in psychiatry. In: Royal College of Psychiatrists London. Available online at: https://www.rcpsych.ac.uk/docs/default-source/training/curricula-and-guidance/general_psychiatry_curriculum_march_2019.pdf.

[24] British Psychological Society. Standards for the accreditation of Doctoral programmes in clinical psychology. Leicester, UK: British Psychological Society (2019).

[25] British Psychological Society. Standards for the accreditation of Doctoral programmes in educational psychology: England, Wales and Northern Ireland. Leicester, UK: British Psychological Society (2019).

[26] Royal College of Psychiatry. Curricula Documents and Resources . Available online at: https://www.rcpsych.ac.uk/training/curricula-and-guidance/curricula-implementation/curricula-documents-and-resources.

[27] Hollingdale J Adamo N Tierney K. Impact of COVID–19 for people living and working with ADHD: A brief review of the literature. AIMS Public Health. (2021) 8:581. doi: 10.3934/publichealth.2021047 34786421 PMC8568596

[28] McGrath J. ADHD and Covid–19: Current roadblocks and future opportunities. Irish J psychol Med. (2020) 37:204–11. doi: 10.1017/ipm.2020.53 PMC7445458 32434606

[29] Lekkos P Thorpe J Obeney–Williams J Xing F. Impact of COVID–19 on referrals for physical and mental health care. BJPsych Open. (2021) 7:S88–9. doi: 10.1192/bjo7_s1

[30] Roughan LA Stafford J. Demand and capacity in an ADHD team: reducing the wait times for an ADHD assessment to 12 weeks. BMJ Open Qual. (2019) 8:e000653. doi: 10.1136/bmjoq-2019-000653 PMC6830462 31750403

[31] Banks J Xu X. The mental health effects of the first two months of lockdown and social distancing during the Covid–19 pandemic in the UK. Fiscal Studies (2020) 41(3):685–708. doi: 10.1111/1475-5890.12239

[32] Montano CB Weisler R. Distinguishing symptoms of ADHD from other psychiatric disorders in the adult primary care setting. Postgraduate Med. (2011) 123:88–98. doi: 10.3810/pgm.2011.05.2287 21566419

[33] Jacob CP Romanos J Dempfle A Heine M Windemuth–Kieselbach C Kruse A. Co–morbidity of adult attention–deficit/hyperactivity disorder with focus on personality traits and related disorders in a tertiary referral center. Eur Arch Psychiatry Clin Neurosci. (2007) 257:309–17. doi: 10.1007/s00406-007-0722-6 17401730

[34] Jensen CM Steinhausen H–C. Comorbid mental disorders in children and adolescents with attention–deficit/hyperactivity disorder in a large nationwide study. ADHD Attention Deficit Hyperactivity Disord. (2015) 7:27–38. doi: 10.1007/s12402-014-0142-1 24942707

[35] Lai MC Lombardo MV Baron–Cohen S. Autism. Lancet. (2014) 383:896–910. doi: 10.1016/S0140-6736(13)61539-1 24074734

[36] Spencer AE Faraone SV Bogucki OE Pope AL Uchida M Milad MR. Examining the association between posttraumatic stress disorder and attention–deficit/hyperactivity disorder: a systematic review and meta–analysis. J Clin Psychiatry. (2016) 77:72–83. doi: 10.4088/JCP.14r09479 26114394

[37] Hansen TF Hoeffding LK Kogelman L Haspang TM Ullum H Sorensen E. Comorbidity of migraine with ADHD in adults. BMC Neurol. (2018) 18:147. doi: 10.1186/s12883-018-1149-6 30322380 PMC6190553

[38] Hirschtritt ME Lee PC Pauls DL Dion Y Grados MA Illmann C. Lifetime prevalence, age of risk, and genetic relationships of comorbid psychiatric disorders in Tourette syndrome. JAMA Psychiatry. (2015) 72:325–33. doi: 10.1001/jamapsychiatry.2014.2650 PMC4446055 25671412

[39] Peleikis DE Fredriksen M Faraone SV. Childhood trauma in adults with ADHD is associated with comorbid anxiety disorders and functional impairment. Nord J Psychiatry. (2022) 76:272–9. doi: 10.1080/08039488.2021.1962973 34392781

[40] Antshel KM Kaul P Biederman J Spencer TJ Hier BO Hendricks K. Posttraumatic stress disorder in adult attention–deficit/hyperactivity disorder: clinical features and familial transmission. J Clin Psychiatry. (2013) 74:e197–204. doi: 10.4088/JCP.12m07698 23561240

[41] Willcutt EG Betjemann RS McGrath LM Chhabildas NA Olson RK DeFries JC. Etiology and neuropsychology of comorbidity between RD and ADHD: the case for multiple–deficit models. Cortex. (2010) 46:1345–61. doi: 10.1016/j.cortex.2010.06.009 PMC2993430 20828676

[42] Blank R Barnett AL Cairney J Green D Kirby A Polatajko H. International clinical practice recommendations on the definition, diagnosis, assessment, intervention, and psychosocial aspects of developmental coordination disorder. Dev Med Child Neurol. (2019) 61:242–85. doi: 10.1111/dmcn.14132 PMC6850610 30671947

[43] Cortese S Moreira–Maia CR St Fleur D Morcillo–Penalver C Rohde LA Faraone SV. Association between ADHD and obesity: A systematic review and meta–analysis. Am J Psychiatry. (2016) 173:34–43. doi: 10.1176/appi.ajp.2015.15020266 26315982

[44] Cortese S Sun S Zhang J Sharma E Chang Z Kuja–Halkola R. Association between attention deficit hyperactivity disorder and asthma: a systematic review and meta–analysis and a Swedish population–based study. Lancet Psychiatry. (2018) 5:717–26. doi: 10.1016/S2215-0366(18)30224-4 30054261

[45] Chen MH Pan TL Hsu JW Huang KL Su TP Li CT. Risk of type 2 diabetes in adolescents and young adults with attention–Deficit/Hyperactivity disorder: A nationwide longitudinal study. J Clin Psychiatry. (2018) 79(3):788–93. doi: 10.4088/JCP.17m11607 29727071

[46] van Rensburg R Meyer HP Hitchcock SA Schuler CE. Screening for adult ADHD in patients with fibromyalgia syndrome. Pain Med. (2018) 19:1825–31. doi: 10.1093/pm/pnx275 29099955

[47] Csecs JLL Iodice V Rae CL Brooke A Simmons R Quadt L. Joint hypermobility links neurodivergence to dysautonomia and pain. Front Psychiatry. (2021) 12:786916. doi: 10.3389/fpsyt.2021.786916 35185636 PMC8847158

[48] Able SL Johnston JA Adler LA Swindle RW. Functional and psychosocial impairment in adults with undiagnosed ADHD. Psychol Med. (2007) 37:97–107. doi: 10.1017/S0033291706008713 16938146

[49] Hamed AM Kauer AJ Stevens HE. Why the diagnosis of attention deficit hyperactivity disorder matters. Front Psychiatry. (2015) 6:168. doi: 10.3389/fpsyt.2015.00168 26635643 PMC4659921

[50] Adamou M Jones SL Fullen T Galab N Abbott K Yasmeen S. Remote assessment in adults with Autism or ADHD: A service user satisfaction survey. PloS One. (2021) 16:e0249237. doi: 10.1371/journal.pone.0249237 33765076 PMC7993762

[51] Good practice in prescribing and managing medicines and devices . Available online at: https://www.gmc-uk.org/-/media/documents/prescribing-guidance-updated-english-20210405_pdf-85260533.pdf.

[52] Giorgi A. The theory, practice, and evaluation of the phenomenological method as a qualitative research procedure. J Phenomenol Psychol. (1997) 28:235–60. doi: 10.1163/156916297X00103

[53] Agrawal N Faruqui R Bodani M. Oxford Textbook of Neuropsychiatry. Oxford: Oxford University Press (2020). doi: 10.1093/med/9780198757139.001.0001

[54] Schneider BC Schöttle D Hottenrott B Gallinat J Moritz S. Assessment of adult ADHD in clinical practice: four letters—40 opinions. J Attention Disord. 2019:1087054719879498. 10.1177/1087054719879498 31625465

[55] Chamberlain SR Cortese S Grant JE. Screening for adult ADHD using brief rating tools: What can we conclude from a positive screen? Some caveats. Compr Psychiatry. (2021) 106:152224. doi: 10.1016/j.comppsych.2021.152224 33581449 PMC7116749

[56] Gelder M Gath D Mayou R Cowen P. Oxford Textbook of Psychiatry, 1996, reprinted 2000. Oxford: Oxford University Press (2000).

[57] Sedgwick JA Merwood A Asherson P. The positive aspects of attention deficit hyperactivity disorder: a qualitative investigation of successful adults with ADHD. ADHD Attention Deficit Hyperactivity Disord. (2019) 11:241–53. doi: 10.1007/s12402-018-0277-6 30374709

[58] Weiss MD McBride NM Craig S Jensen P. Conceptual review of measuring functional impairment: findings from the Weiss Functional Impairment Rating Scale. Evid Based Ment Health. (2018) 21:155–64. doi: 10.1136/ebmental-2018-300025 PMC6241626 30314990

[59] Silveira C Souza RT Costa ML Parpinelli MA Pacagnella RC Ferreira EC. Validation of the WHO Disability Assessment Schedule (WHODAS 2.0) 12–item tool against the 36–item version for measuring functioning and disability associated with pregnancy and history of severe maternal morbidity. Int J Gynecol Obstet. (2018) 141:39–47. doi: 10.1002/ijgo.2018.141.issue-S1 PMC6001571 29851113

[60] Goodman DW Surman CB Scherer PB Salinas GD Brown JJ. Assessment of physician practices in adult attention–deficit/hyperactivity disorder. Primary Care Companion CNS Disord. (2012) 14(4). doi: 10.4088/PCC.11m01312 PMC3505127 23251858

[61] SASC Guidance on the assessment and identification of the characteristics of an Attention Deficit Hyperactivity Disorder (ADHD) . Available online at: https://www.patoss-dyslexia.org/write/MediaUploads/ADHD_Guidance_JUNE_21.pdf.

[62] ADHD assessment system 'broken' with five–year waiting times . Available online at: https://www.bbc.com/news/uk-england-53526174 (Accessed 13 June 2024).

